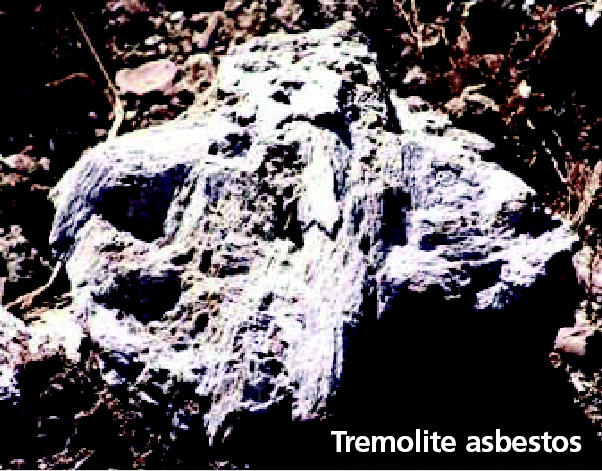# Asbestos: Showdown in El Dorado

**Published:** 2004-11

**Authors:** Rebecca Renner

Taken at face value, northern California’s El Dorado County has a lot going for it—the dramatic Sierra Nevada foothills scenery, lots of room for spacious new houses, and its short distance from Sacramento. That’s why the population has nearly sextupled since 1960, according to U.S. Census figures. But the construction that is transforming this once rural area has also dug up a health risk—thin needles of amphibole asbestos, a particularly hazardous form of the mineral.

Naturally occurring amphibole asbestos is not a problem when left underground. But development can unearth the mineral, increasing the risk of exposure. In November 2003 the U.S. Environmental Protection Agency (EPA) found that over 25% of 153 soil samples collected from the local high school contained more than 1% asbestos by weight, a level that, if disturbed, could pose a threat to public health. As a result, the agency determined that additional investigations are required. This fall, officials from the EPA Superfund program plan to simulate the activities of school kids playing sports and use personal monitors to measure exposures associated with these activities at several locations including the high school, according to Superfund senior science advisor Richard Troast.

The monitoring comes in the wake of another town’s recently publicized experience: occupational and environmental exposure to amphibole asbestos in the small mining town of Libby, Montana, resulting in deaths and other widespread health effects. There are lessons from Libby that apply to El Dorado, according to many asbestos experts. People with no occupational exposure, such as women who handled their miner husbands’ work clothes, can have asbestos-related problems. Further, ambient air monitoring may not reflect actual asbestos exposure—individuals can dramatically heighten their exposure by kicking up fibers on the ground. So an accurate estimate of exposure requires personal air monitoring during specific activities.

In the April 2004 issue of *Occupational and Environmental Medicine*, a group of British researchers led by J. Corbett McDonald reported their study of 406 Libby miners, in which they calculated that occupational exposure resulted in a 14% increase in mortality from all asbestos-related causes. They also estimated that environmental exposure for 50 years would lead to a 3.2% mortality increase and called attention to the potential health risk in northern California. Extrapolation is fraught with uncertainty, but for El Dorado’s population of 160,000, such a mortality increase could translate into thousands of deaths.

A 2002 EPA peer consultation unanimously agreed that for mesothelioma, a rare cancer of the lining of the lung, the carcinogenic potency of amphibole fibers is a minimum of two orders of magnitude greater than for chrysotile asbestos fibers (most likely because amphibole fibers persist longer in the body). Forms of amphibole asbestos also can cause noncancer diseases in proportion to exposure, according to researchers led by Lucy Peipins of the Agency for Toxic Substances and Disease Registry. They conducted a clinical study in which about 6,700 Libby residents had chest X rays. In the November 2003 issue of *EHP*, the researchers reported 29 exposure pathways related to work, recreation, and household activities. The prevalence of pleural abnormalities increased with the number of exposure pathways, ranging from 6.7% for those who reported no apparent exposures to 34.6% for those who reported 12 pathways.

Some data already indicate that exposures in El Dorado are high enough to be harmful. When concerned citizens requested analysis of four deceased pets, the animals’ lung fiber burdens revealed concentrations of amphibole fibers higher than those found in goats from Corsica, where episodic environmental exposure to amphibole asbestos is clearly associated with human mesothelioma, says pathologist Jerrold Abraham of Upstate Medical University. Working collaboratively, pathologist Bruce Case of McGill University confirmed these findings in an independent analysis. A summary of the study findings is available online at **http://www.asbestos.net/**.

What happens next in El Dorado will depend on the exposure monitoring data, says Troast. Some experts are braced for the worst. Says Case, “The situation in El Dorado has the potential to be the most important source of environmental asbestos-related mesothelioma ever in the United States.”

## Figures and Tables

**Figure:**